# Addressing methodological limitations in the comparison of robotic surgical stress responses: a call for broader context and enhanced biomarker profiling

**DOI:** 10.1097/JS9.0000000000004671

**Published:** 2026-01-13

**Authors:** Haoxiang Qin, Zhihang Fan, Xiaoyu Yang, Bin Ma

**Affiliations:** a Department of Colorectal Surgery, Cancer Hospital of China Medical University, Cancer Hospital of Dalian University of Technology, Liaoning Cancer Hospital and Institute, Shenyang, China

*To the Editor,*

We read with great interest the article by Liu *et al*, which presented a post hoc analysis comparing the surgical stress response between the Kangduo (KD) and da Vinci (DV) robotic systems in colorectal cancer surgery[1]. The authors are to be commended for their work in providing physiological data on an emerging robotic platform. However, we wish to raise two critical methodological limitations that, in our view, constrain the broader clinical interpretation of the findings and suggest important avenues for future research. In line with the TITAN 2025 guideline on transparent reporting of artificial intelligence in surgical research[2], we explicitly state that artificial intelligence tools were used only for language polishing during the preparation of this correspondence.

First, the study’s exclusive focus on a head-to-head comparison between two robotic systems, while valuable, leaves a significant contextual gap. The demonstrated non-inferiority of KD to DV in stress marker trajectories is reassuring for technological parity. However, this design inherently cannot address the fundamental question of whether robotic-assisted surgery itself confers a physiological advantage over conventional approaches. Laparoscopic surgery is the established minimally invasive standard for colorectal resection, proven to elicit a attenuated stress response compared to open techniques[3]. Where, then, does the robotic modality fit within this spectrum? Does its enhanced dexterity and precision translate into a measurably lower inflammatory burden or superior immune preservation than laparoscopy? A comparative trial by Cuk *et al* directly measured stress responses between robotic and laparoscopic colon cancer surgery, finding lower C-reactive protein levels postoperatively in the robotic group[4]. Without a laparoscopic or open surgery control arm, the study by Liu *et al* cannot confirm if the stress responses observed with either robotic system are meaningfully superior to those of the current standard of care. Consequently, the clinical and economic rationale for adopting robotic systems – especially newer platforms – remains partially substantiated from a physiological standpoint. Future studies incorporating a three-arm design (robotic, laparoscopic, and open) are essential to delineate the specific incremental benefit, if any, of robotic assistance on surgical trauma and recovery[5].

Second, the selection of stress biomarkers, though covering hepatic, renal, and hematological parameters, appears notably narrow regarding the core inflammatory and immune response. The study quantified C-reactive protein and neutrophil-to-lymphocyte ratio but omitted pivotal cytokines such as interleukin-6 (IL-6), a primary driver of the acute-phase response and a sensitive marker of surgical trauma[6]. Similarly, interleukin-10, an anti-inflammatory cytokine, and procalcitonin, a specific marker associated with bacterial infection and systemic inflammation, were not assessed. Can a comprehensive appraisal of postoperative immune modulation be made without measuring these key mediators? The omission is consequential. Robotic surgery has been hypothesized to better preserve immune function due to reduced tissue handling; this claim necessitates evaluation through direct measurement of Th1/Th2 cytokine profiles[7]. Furthermore, procalcitonin could provide crucial differentiation between sterile surgical stress and early infectious complications, a common confounding factor in postoperative biomarker studies. Relying on conventional markers alone risks overlooking subtler but clinically significant immunologic advantages one platform may have over another. Future investigations would be greatly strengthened by incorporating a broader panel of biomarkers, including dynamic cytokine profiling, to more fully capture the complexity of the surgical stress response and its modulation by different surgical techniques.

In conclusion, while the study by Liu *et al* successfully establishes equipoise between the KD and DV systems, its design limits our understanding of how robotic stress responses compare to conventional surgery and whether the chosen biomarkers fully reflect the underlying immunologic state. Addressing these points in future research will be vital for positioning robotic platforms within the broader surgical armamentarium and for truly evaluating their physiological impact on patient recovery.

## Data Availability

No new data were generated or analyzed in this correspondence. Data sharing is not applicable to this article.
